# Giant mucinous borderline ovarian tumor: challenges of diagnosis and treatment

**DOI:** 10.1007/s00404-024-07793-8

**Published:** 2024-10-23

**Authors:** Pawel Sadlecki, Katarzyna Dejewska, Patrycja Domieracka, Malgorzata Walentowicz-Sadlecka

**Affiliations:** 1 Medical Department, University of Science and Technology, Bydgoszcz, Poland; 2 Department of Obstetrics, Gynecology and Gynecologic Oncology, Regional Polyclinical Hospital, Grudziadz, Poland; 3 Department of Obstetrics Gynecology and Gynecologic Oncology, Regional Polyclinical Hospital, Torun, Poland

Dear Editor,

Sixty-two-year-old patient presented to our Department with moderate abdominal pain and a significantly enlarged abdominal circumference. An abdominal CT scan detected a giant polycyclic fluid space, with a solid area originating from the adnexa (Fig. 1A). Tumor markers showed CA 125 at 79.62 U/mL and HE4 at 123.9 pmol/l. Surgical treatment involved a median suprapubical incision extended beyond the umbilicus. The tumor originating from the left adnexa was excised (Fig. 1B). Both the uterus and the remaining adnexa were removed. No hemodynamic or cardiac complications were observed intraoperatively. The final histopathological examination revealed a tumor measuring 40 cm and weighing 14.46 kg. The tumor was diagnosed as a borderline malignant mucinous tumor of the ovary (Fig. 1C). Postoperative recovery was uneventful, and the patient was discharged on the fourth day after surgery.

**Fig. 1:**
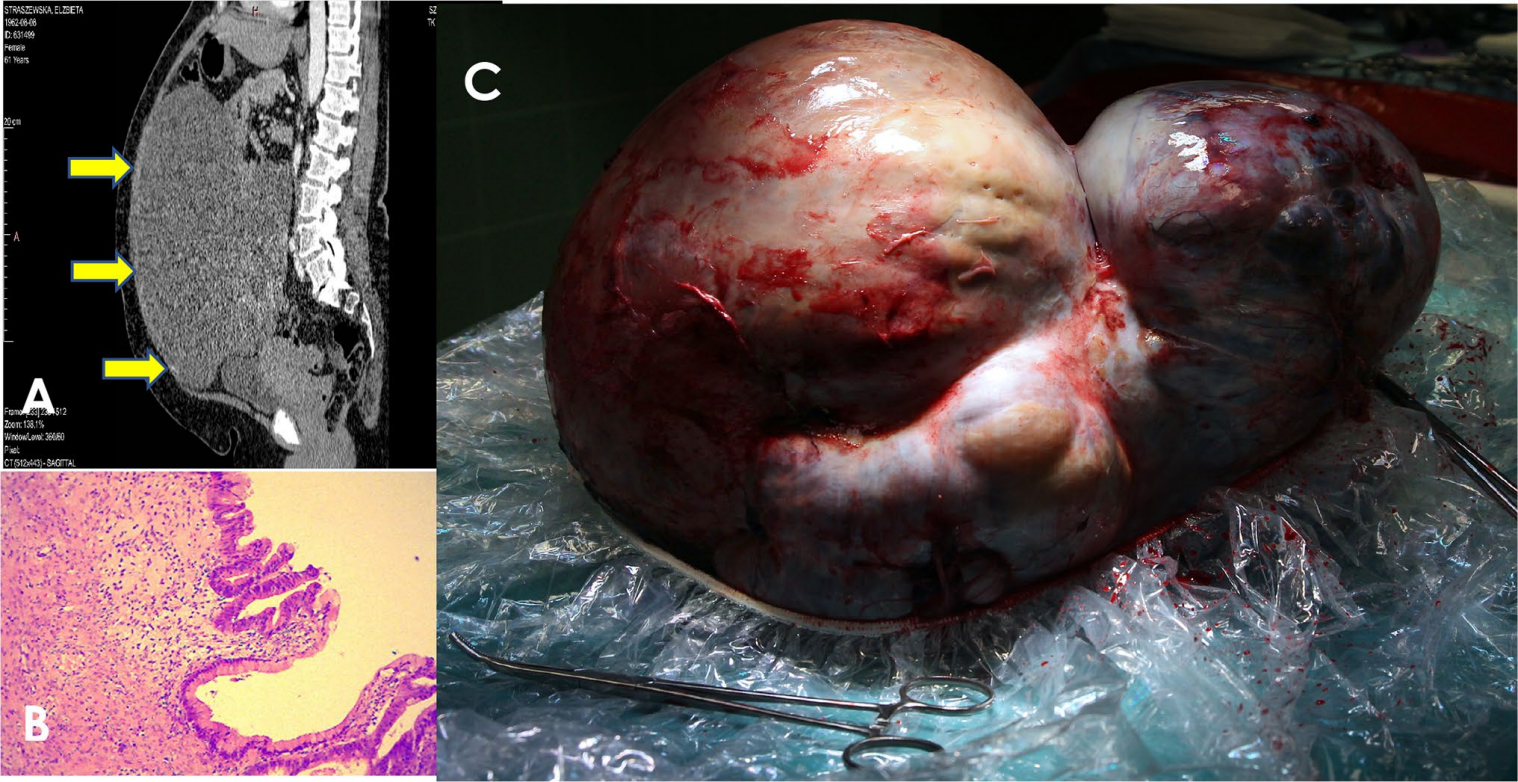
**A** Abdominal CT scan detected a polycyclic fluid space 40 cm in length originating from the adnexa (arrows). **B** **H&E (Hematoxylin and Eosin)** stained specimens of mucinous borderline ovarian tumor, primary objective magnification 10x. **C** The tumor was removed without any spillage with intact outer wall

## Discussion

The diagnosis of giant borderline ovarian tumor (gBOT) can be established through physical examination, USG, CT, or MRI [1]. Despite these diagnostic tools, the possibility of malignancy often remains uncertain until a final pathologic diagnosis is made. Treatment strategies are guided by the FIGO classification, age and the patient’s fertility wishes. For patients with a FIGO grade I tumor (confined to the ovary): If fertility preservation is not a concern, a hysterectomy with bilateral salpingo-oophorectomy may be performed. If the patient wishes to preserve fertility, a unilateral salpingo-oophorectomy can be applied [2]. A critical aspect of surgical treatment is ensuring complete removal of the lesion while avoiding rupture of the tumor capsule during surgery which can increase the risk of disease progression and recurrence [3]. The literature underscores the significant risk of intraoperative complications due to rapid changes in body circulation, which include pulmonary and cardiac failure or pulmonary embolism [4]. The complexity of treating gBOTs necessitates a multidisciplinary approach, which is crucial for providing optimal patient care.

## Data Availability

The data used to support the findings of this study are available from the corresponding author upon request.
